# Genetic ancestry and genotype-by-diet interactions shape plasma carotenoid metabolism in humans

**DOI:** 10.21203/rs.3.rs-11145064/v1

**Published:** 2026-09-28

**Authors:** Yixing Han, Savannah Mwesigwa, Qiang Wu, Stephanie B. Jilcott Pitts, Nancy E. Moran, Neil A. Hanchard

**Affiliations:** 1) Center for Precision Health Research, National Human Genome Research Institute, National Institutes of Health, Bethesda, MD, United States; 2) Department of Medical Microbiology, College of Health Sciences, Makerere University, Kampala, Uganda; 3) Department of Public Health, East Carolina University, Greenville, NC, United States; 4) USDA/ARS Children’s Nutrition Research Center, Department of Pediatrics, Baylor College of Medicine, Houston, TX, United States; 5) Department of Molecular and Human Genetics, Baylor College of Medicine, Houston, TX, United States

**Keywords:** genotype-diet interaction, plasma carotenoids, multi-ancestry, precision nutrition, nutrient metabolism, heritability, GWAS

## Abstract

Interindividual variation in circulating nutrients reflects intersections of diet, ancestry and host genetics, yet genotype-diet regulation of nutrient metabolism remains difficult to map in diverse populations. Here, we integrate multi-ancestry genome-wide genotyping and targeted sequencing with a longitudinal randomized dose-response trial to study species-specific plasma carotenoid metabolism. Using plasma carotenoid concentrations as integrated readouts in two deeply phenotyped, multi-ancestry U.S. cohorts: a discovery cohort (n=213) and an intervention/replication cohort (n=162), we show that SNP-based heritability varies substantially across carotenoid species and ancestry groups (h^2^=0.08–0.44), with plasma β-cryptoxanthin exhibiting the highest estimate and ancestry-dependent enrichment. GWAS identified β-cryptoxanthin associated loci at 3q13 (*IGSF11* region, rs80316816, P=6.25×10–10, β=0.74) and 19p13 (*R3HDM4/MED16* region, rs28468554, P=6.26×10–9, β=−0.46), collectively explaining over 30% of β-cryptoxanthin variance in the discovery cohort. Crucially, in the intervention cohort, 3q13 variants showed genotype-dependent, dose-specific effects on β-cryptoxanthin response to supplementation. Targeted sequencing implicated the *PKD1L2-BCO1* locus for β-carotene (Padj=0.04, β=−1.3 to −0.3), and rare predicted-to-be-damaging variants in carotenoid- and lipid-related genes, including *BCO1*, *CETP* and *APOA1*, among individuals with extreme carotenoid concentration phenotypes. Together, these findings show how genetic diversity and genotype-diet interactions shape diet-responsive nutrient metabolism, advance a framework for precision nutrition genetics.

## INTRODUCTION

Blood nutrient and phytochemical concentrations are key predictors of nutritional adequacy and disease risk. However, human variability in dietary nutrient and phytochemical metabolism arises from differences in dietary exposure, host physiology, and genetic variation. Precision nutrition seeks to explain this metabolic heterogeneity by identifying biologically meaningful subgroups that differ in metabolic response, rather than assuming uniform dietary requirements or responses across populations^1^. Consistent with this framework, controlled and real-world studies show that individuals can exhibit markedly different metabolic responses to similar foods, underscoring the need to understand the mechanisms driving diet-dependent variation^2–4^. However, the genetic determinants of nutrition and phytochemical metabolism, particularly genotype-diet interaction, remain incompletely characterized, especially in ancestrally diverse populations^5,6^.

Plasma concentrations of carotenoids, red, orange, and yellow antioxidant phytochemicals found predominantly in fruits and vegetables, are associated with a reduced risk for chronic diseases including some cancers, stroke, and myocardial infarction^7^. Yet, as with other nutrients and phytochemicals, blood carotenoid concentrations are not solely predicted by dietary intake^7^. Plasma carotenoid concentrations provide a tractable model for studying genetic regulation of dietary phytochemical metabolism because their circulating concentrations integrate diet intake, intestinal absorption, lipid transport, enzymatic conversion and tissue distribution^7^. While there are hundreds of carotenoid species identified in nature^8^, six species (α-carotene, β-carotene, β-cryptoxanthin, lycopene, lutein, and zeaxanthin) are predominant in the food supply and constitute more than 95% of total human blood carotenoids^9^. Plasma carotenoid concentrations correlate with fruit and vegetable intake, with specific food groups explaining meaningful variance in particular carotenoid species (for example, fruit intake is a strong predictor of plasma β-cryptoxanthin), supporting their utility as objective nutritional biomarkers^10^. However, clinical studies repeatedly observe persistent interindividual variability in carotenoid absorption and circulating/tissue responses, even under similar intake, indicating a significant contribution from host-intrinsic factors^7,11^.

The biological pathways underlying carotenoid metabolism provide multiple points at which genetic variations could influence circulation concentrations. Carotenoid processing overlaps with lipid and cholesterol metabolism: carotenoids are absorbed and transported via pathways involving uptake and transport proteins (*SR-BI/SCARB1, CD36, NPC1L1*) and cleavage enzymes (*BCO1/BCMO1, BCDO2*), providing multiple plausible routes for genetic variation to influence carotenoid bioavailability and circulating levels^7,11^. In addition, diet and genetics can interact directly within these pathways. For example, experimental work has demonstrated that the intestine-specific transcription factor *ISX* regulates *BCMO1 (BCO1)* expression and links nutritional state (retinoic acid levels and signaling) to genetic control of provitamin A conversion, providing a concrete biological precedent for genotype-dependent nutrient metabolism^12^.

The discovery genetic loci that influence plasma carotenoid concentrations, however, has been limited such that most of the existing studies have focused on European-ancestry cohorts, thereby reducing generalizability and missing ancestry-informative architecture relevant to metabolic traits^5,6^. Early genome-wide studies identified common variation near *BCMO1* was associated with circulating carotenoid levels, establishing that common genetic variants can measurably shape carotenoid status^13^. However, the extent to which genetic ancestry modifies carotenoid heritability, locus discovery, and dietary response remine poorly understood. More broadly, trans-ethnic genetic study designs can improve discovery and characterization of trait loci by leveraging differences in allele frequency and linkage disequilibrium across populations, aiding replication and fine mapping^14,15^.

Here, we leverage two deeply phenotyped, ancestrally and geographically diverse U.S. cohorts to characterize the genetic architecture of total- and species-specific plasma carotenoids. We estimated SNP-based heritability across ancestry groups, identified and replicated common variant associations via genome-wide analysis, complemented by targeted sequencing of carotenoid metabolism genes, and tested genotype-dependent dietary responsiveness within a randomized dietary dose-response intervention. Collectively, these analyses use plasma carotenoid concentrations as an integrated readout of nutrient metabolism to identify ancestry-dependent and diet-responsive genetic influences on carotenoid status in humans, which may ultimately explain variability in healthy responses to dietary carotenoids.

## METHODS

### Study design and carotenoid species measurement

Plasma carotenoid concentration and dietary data were obtained from two previously published U.S. cohorts that were deeply phenotyped for dietary intake and carotenoid biomarkers^16,17^. The Primary Study Cohort was a cross-sectional observational study of healthy adults aged 18–65 years recruited from North Carolina and Minnesota (n=213 enrolled; n=207 included after genetic QC and relatedness filtering; **Supplementary Table S1**)^16^. The Intervention Cohort was a 6-week randomized dietary carotenoid dose-response trial recruited from North Carolina, Minnesota, and Texas (n=162; **Supplementary Table S2**)^17^ including 110 participants who were not included in the Primary Study Cohort and were therefore used for replication analyses. Participants in the Intervention Cohort were randomized to receive a daily, carotenoid-rich juice providing 0 mg/day (control), 4 mg/day (moderate), or 8 (high) mg/day total carotenoids, with plasma carotenoid concentrations measured at baseline (week 0), week 3, and week 6. Plasma concentrations of total carotenoids and five major carotenoid species were quantified by HPLC with photodiode-array detection^17^. Dietary intake was assessed using self-administered repeated 24-hour dietary recalls, and daily intervention adherence was recorded^17^. Both studies were approved by the East Carolina University Medical Center institutional review board (17–001242). The randomized trial was registered (NCT04056624), and all participants provided written informed consent.

### Genotyping, quality control, and imputation

Genome-wide genotyping was performed in both cohorts using the Infinium H3Africa Consortium Array v2 (Illumina). Sample-level and variant-level quality control (QC), including call rate, sex checks, heterozygosity outliers, and removal of related individuals, was performed using standardized parameters (**Supplementary Methods**). After QC, 1,917,156 autosomal genotyped SNPs were retained in 207 unrelated individuals. Genotypes were phased and imputed using the Michigan Imputation Server^18^ with the 1000 Genomes Project Phase III v5 reference panel^19^ on the GRCh37/hg19 build. Imputed variants were filtered by imputation quality (r^2^≥0.3) and minor allele frequency (MAF≥0.05), yielding 7,467,403 SNPs for genome-wide association analysis.

### Genetic ancestry inference

To infer genetic ancestry independent of self-reported race/ethnicity, cohort genotypes were merged with 1000 Genomes Phase III reference samples^19^ after removing strand-ambiguous SNPs (A/T and C/G) and performing linkage disequilibrium pruning (PLINK --indep-pairwise 50 10 0.1). Multidimensional scaling (MDS) was performed in PLINK to derive ancestry axes, and participants were aligned to the nearest 1000 Genomes superpopulation clusters: African (AFR), Admixed American (AMR), East Asian (EAS), European (EUR), South Asian (SAS). Genetically inferred ancestry was used for ancestry-stratified summaries and heritability analyses, whereas MDS1 and MDS2 were included as continuous covariates in association models. Self-reported race/ethnicity group was retained for descriptive presentation only.

### SNP-based heritability

SNP-based heritability (h^2^) for total plasma carotenoids and individual carotenoid species was estimated using Genome-wide Complex Trait Analysis (GCTA v1.94.1)^20^ with restricted maximum likelihood. A genomic relationship matrix was constructed from directly genotyped, QC-filtered autosomal genotyped SNPs with MAF≥0.01. Imputed variants were not used for heritability estimation to avoid introducing imputation uncertainty into the genomic relationship matrix. Models included age, sex, BMI, study site, and the first two MDS dimensions as covariates (**Supplementary Methods**). Relative heritability for each ancestry cluster was defined as the within-cluster heritability estimate divided by the full-cohort heritability for the same phenotype.

### Genome-Wide Association Studies (GWAS)

Genome-wide association studies (GWAS) were conducted using GEMMA (v0.98.5)^21^ under a linear mixed model to account for cryptic relatedness, with the kinship matrix estimated from genotyped SNPs. Plasma carotenoid phenotypes and dietary carotenoid intake were log2-transformed to improve normality (Shapiro–Wilk test; **Supplementary Table S3**). Association models included age, sex, BMI, study site, log2 dietary carotenoid intake, and MDS1–2 as covariates. Genome-wide significance was defined as P<5×10^−8^ and suggestive association was defined as P<1×10^−6^. As a sensitivity analysis, lead GEMMA signals were re-tested using PLINK v1.9 with the same covariates where applicable; concordance in direction and approximate effect magnitude was used as a robustness check and was not used to define lead variants.

### Replication, meta-analysis, and genotype-by-dose interaction

For replication, we tested lead variants from loci reaching genome-wide significance or suggestive association in the Primary Study Cohort in the subset of Intervention Cohort participants not included in the discovery cohort (n=110) using the same covariate-adjusted GEMMA model framework used in the Primary Study Cohort. Fixed-effects meta-analyses across cohorts were performed in METAL^22^ using inverse-variance weighting of effect estimates and standard errors.

To evaluate genotype-dependent dietary responsiveness, we modeled week 6 log2-transformed plasma carotenoid concentrations in the Intervention Cohort using linear regression. Predictors include additively coded genotype (0/1/2 effect alleles), randomized intervention dose coded 0, 1, or 2 corresponding to 0, 4, or 8 mg/day total carotenoids, baseline (week 0) log2-transformed plasma carotenoid concentration, and covariates including age, sex, BMI, study site, and MDS1–2. A genotype × dose interaction term was included to test whether genotype modified the plasma carotenoid response to increasing supplementation dose. Given limited power for interaction testing in this cohort, nominal interaction P-values are reported, and loci with P_genotype × dose_ <0.1 were prioritized for visualization.

### Targeted sequencing and variant processing

To improve coverage of candidate carotenoid metabolism loci that may be incompletely captured by arrays genotyping and imputation, particularly in diverse populations^23,24^, we performed targeted sequencing of 35 carotenoid metabolism-related genes selected from the literature (**Supplementary Table S7**) using an Illumina Ampliseq Custom Panel. Reads were aligned to the GRCh38/hg38 reference genome using BWA-MEM^25^ with the following parameters: -M -O 30 -E 4 -T 20 -v 3. BAMs were then merged and indexed using SAMtools (v1.5)^26^. Subsequently, variant calling followed the Genome Analysis Toolkit (GATK v4)^27–29^ Best Practice including duplicate marking, base quality score recalibration, HaplotypeCaller in GVCF mode, and joint genotyping. Variant annotation was performed using ANNOVAR^30^ (version 2020-06-08). Per-base coverage was computed with bedtools^31^ coverage (**Supplementary Table S7, Supplementary Figure S6**).

### Targeted association testing and rare-variants analysis

Variants within the AmpliSeq targeted regions were filtered using the Variants Association Tool^32^ and VCFTools (v0.1.15)^33^. Within targeted regions, variants were retained for association testing if they had call rate ≥95%, Hardy–Weinberg equilibrium P≥1×10^−3^, and MAF≥0.01. Associations between targeted variants and plasma carotenoid phenotypes were evaluated using linear regression models adjusted for the same covariates as GWAS. P-values were corrected for multiple testing using the Benjamini–Hochberg false discovery rate procedure (Padj).

To identify potentially high-impact rare variants, we screened individuals with extreme plasma carotenoid phenotypes (>2 SD above or below the cohort mean) for rare (gnomAD MAF<0.01) protein-altering variants, including missense, stop-gain, or essential splice-site. Prioritized variants were annotated with in silico deleteriousness scores (e.g., CADD and AlphaMissense) (**Supplementary Methods**).

### Software and statistical analysis

Unless otherwise specified, statistical analyses were performed using R v4.3 and reported P-values are two-sided. Additional implementation details, quality-control thresholds, and software parameters are provided in the Supplementary Methods.

## RESULTS

### Cohort characteristics

Genetic analyses were conducted in two extensively characterized, previously described, ancestrally diverse cohorts^16,17^ (Methods). The Primary Study Cohort serves as the discovery cohort and comprised 213 adults, of whom 207 remained after QC and familial relatedness filtering. Participants were self-reported as four racial and ethnic groups: Asian, Hispanic, Non-Hispanic Black, and Non-Hispanic White (**Supplementary Table S1**). Plasma carotenoid species concentrations were measured by HPLC-photodiode array detection, and participants were genotyped on the H3Africa genotyping microarray. Targeted sequencing of 35 carotenoid metabolism related loci complemented array coverage (Methods, **Supplementary Table S7**).

The Intervention Cohort consisted of 162 individuals, of whom 110 were non-overlapping with the Primary Study Cohort, **Supplementary Table S2**) recruited from three sites in a 6-week randomized carotenoid-rich juice dose-response trial^17^ and were used for replication of discovery association signals. Plasma carotenoids were measured at weeks 0, and at weeks 3 and 6, post-intervention, across control (0 mg/d), moderate (4 mg/d), and high (8 mg/d) total carotenoid doses groups. Participants were genotyped using the same platform, and targeted sequencing of carotenoid metabolism-related genes were performed to complement array-based analysis (Figure 1) (Methods and **Supplementary Methods**).

### Genetic ancestry structure and descriptive plasma carotenoid variation

Because U.S. race/ethnicity categories do not directly map to genetic ancestry, we inferred genetic ancestry by projecting cohort genotype on to 1000 Genomes Phase III reference superpopulations^19^ using multi-dimensional scaling (MDS) (Figure 2A). Most Non-Hispanic White and Non-Hispanic Black participants clustered closely with the European (EUR) and African (AFR) reference groups, respectively, and self-identified Hispanic participants clustered with the Admixed American (AMR) reference group. Self-identified Asian participants separated into two distinct clusters aligning with South Asian (SAS) and East Asian (EAS) reference groups (Figure 2A). A small number of individuals did not cluster with the majority genetic ancestry of their self-reported race/ethnicity group (Figure 2A). For downstream genetic analyses, genetically inferred ancestry and MDS covariates were used rather than self-reported race/ethnicity.

Plasma carotenoid concentrations are influenced by a variety of factors, including variations in host-intrinsic biology, some of which has been loosely related to self-reported racial/ethnic background^16,34^ (Figure 2B, 2C; **Supplementary Figure S2**), but may be more strongly related to genetic ancestry. Overall, differences in plasma carotenoids across the four race/ethnicity groups in the observational, Primary Study Cohort were modest (ANOVA); however, several pairwise comparisons, particularly between Non-Hispanic Black and Asian participants, showed significant differences, including concentrations of total plasma carotenoids (Welch’s Two-Sample *t*-tests P = 0.021), β-carotene (P = 0.003), and lutein/zeaxanthin (P = 0.037) (Figure 2; Supplementary Figure S2). Plasma β-cryptoxanthin concentrations differed nominally between genetically inferred EAS and SAS participants (P=0.027, **Supplementary Figure S3**), whereas other carotenoid species did not show significant EAS-SAS differences. Given the modest sizes of these subgroups (EAS n=21, SAS n=31), this observation should be interpreted cautiously until evaluated in larger cohorts. These descriptive comparisons do not account for dietary intake or other biological covariates; they were used as context for subsequent ancestry-aware genetic analyses rather than as primary evidence of genetic effects.

### Plasma carotenoids exhibit species-specific and ancestry-dependent heritability

Using directly genotyped, QC-filtered SNPs from 207 unrelated individuals (1,917,156 autosomal SNPs, MAF≥0.01; Methods), we estimated SNP-based heritability of total and species-specific plasma carotenoid concentrations with GCTA^20^(**Supplementary Table S4**; Methods). Imputed variants were not used for heritability estimation to avoid imputation uncertainty. Total plasma carotenoid showed overall low SNP heritability (h^2^=0.08, se= 0.157), consistent with strong dietary contribution from dietary exposure and differential metabolism of its constituent subspecies. Accordingly, heritability varied substantially across plasma carotenoid species: ranging from the lowest estimated heritability for lutein/zeaxanthin (h^2^=0.09, se= 0.172), to the highest estimated heritability for β-cryptoxanthin (h^2^=0.44, se=0.322) (Figure 3A, Supplementary Table S4).

To better understand ancestry-related differences in genetic contribution to carotenoid phenotypes, we estimate relative heritability across genetically inferred ancestry groups. Given the ancestry-stratified subgroups (n=21–70), these estimates had large standard errors and were interpreted descriptively. Relative heritability for total plasma carotenoids was generally similar across groups, except among AMR-clustering participants, where the estimate was higher than in the full cohort. Similar patterns were observed for plasma α-carotene, β-carotene, and total lycopene, among AMR-clustering participants. By contrast, β-cryptoxanthin heritability was enriched (2.3x) among AFR-clustering participants, while the lycopene heritability was notably higher among SAS-clustering participants (2.4x) (Figure 3B).

### Genome-wide analyses identify and replicate novel loci for plasma carotenoid subspecies

Given the species-specific heritability patterns, we next sought to identify genetic loci associated with total and species-specific plasma carotenoid concentrations across our cohort. GWAS of total and species-specific plasma carotenoid concentrations were conducted using GEMMA linear mixed models with log_2_-transformed phenotypes, adjusting for age, sex, BMI, log2-transformed dietary carotenoid intake, and MDS1–2 (Methods; **Supplementary Table S3**). The MDS covariates were included to reduce confounding from global population structure while preserving locus-specific genotype effects.

We identified six genome-wide significant SNPs across three loci (P < 5×10^−8^), one SNP at the *RAPGEF1* locus on chromosome 9 associated with α-carotene, three SNPs at the 3q13 locus associated with β-cryptoxanthin, and two SNPs at the 19p13 locus associated with β-cryptoxanthin (Table 1, Figure 4). An additional 37 SNPs across 12 loci surpassed a suggestive association threshold (P < 1×10^−6^) (**Supplementary Table S5**). The most robust signals were observed for β-cryptoxanthin at loci on 3q13 and 19p13. The α-carotene association was led by rs3765544, located in intron 24 of the *RAPGEF1* gene, on chromosome 9q34 (P = 8.86 × 10^−10^, beta = 0.75; Table 1, **Supplementary Figures S4**), corresponding to ~68% higher α-carotene concentrations per effect allele (2^β). *RAPGEF1* encodes a guanine nucleotide exchange factor involved in the activation of *Ras* family GTPases^35,36^ that plays a role in multiple cellular signaling pathways^37,38,39^. Notably, the same allele also showed marginal association with β-carotene levels (P = 6.43 × 10^−8^, beta = 0.79; Table 1, **Supplementary Figure S4, Supplementary Table S5**), suggesting that this locus may influence more than one provitamin A carotenoid phenotype.

To evaluate replication of our primary cohort findings, we tested discovery lead variants in the 110 Intervention Cohort participants who did not participate in the Primary Study Cohort, using baseline plasma carotenoid measurements and the same covariate-adjusted model framework used in the Primary Study Cohort (Methods). Fixed effect meta-analysis of candidate SNPs across the two cohorts strengthened the evidence for the association between 3q13 and β-cryptoxanthin across cohorts. In particular, rs76613159 found near *IGSF11* reached genome-wide significance in the meta-analysis (P_meta=1.62×10^−8^, β=0.71), and multiple nearby variants showed concordant effects (Table 1; **Supplementary Table S5**). The 19p13 locus showed consistent direction but weaker statistical support in the meta-analysis (**Supplementary Table S5**).

### GWAS-identified loci associated with β-Cryptoxanthin phenotypes

Consistent with the high heritability of plasma β-cryptoxanthin, we observed two genome-wide significant loci associated with β-cryptoxanthin concentrations. At chromosome 3q13, the strongest signals mapped at downstream of *IGSF11* with lead variants including rs80316816 (P=6.25 × 10^−10^, beta=0.74), rs76613159 (P=4.95 × 10^−9^, beta=0.71), and rs76087842 (P=8.32 × 10^−9^, beta = 0.72) (Figure 4A, 4B, Table 1). At 19p13, the strongest signals mapped near *R3HDM4/MED16* with top SNPs: rs28468554 (P=6.26 × 10^−9^, beta=−0.46) (Figure 4A, 4C, Table 1). Collectively, these two loci accounted for ~33% of β-cryptoxanthin variance in our discovery cohort.

### Variants at 3q13 show genotype-dependent dietary responsiveness to carotenoid supplementation

To test whether β-cryptoxanthin-associated variants also influenced response to controlled carotenoids intake, we evaluated genotype-by-dose interaction in the Intervention Cohort. Using longitudinal measurements, we built models predicting week 6 plasma β-cryptoxanthin concentrations while adjusting for baseline β-cryptoxanthin, randomized dose group, genotype, and covariates (Methods). Six variants at 3q13, of which five are upstream of *IGSF11* showed suggestive genotype-by-dose interactions (Pgenotype × dose < 0.1) (Figure 5; **Supplementary Table S6, Supplementary Figure S6**). These variants showed genotype-dependent dietary responsiveness of plasma β-cryptoxanthin, supporting 3q13 as a candidate diet-responsive locus for β-cryptoxanthin metabolism. These variants showed dose-dependent divergence in β-cryptoxanthin response by genotype, supporting 3q13 as a candidate diet-responsive locus for β-cryptoxanthin metabolism.

### Targeted sequencing identifies rare variants association at the *PKD1L2-BCO1* locus for β-carotene

Array-based genotyping can provide sparse coverage at some established carotenoid metabolism loci, particularly in diverse populations^23,40^, so we performed targeted sequencing of 35 carotenoid-metabolism-related genes identified from the literature^7,11,24^ (**Supplementary Table S7**). Our targeted capture was designed to encompass gene coding regions and thus did not directly sequence previously reported SNPs upstream of *BCO1*^13,41^; however, patterns of recombination and linkage disequilibrium (LD) at the locus suggest that *PKD1L2* variants are likely to be in the same LD block. Targeted sequencing revealed associations between variants at the *PKD1L2–BCO1* locus and plasma β-carotene concentration (Padj = 0.04; β = −1.3 to −0.3), supporting an effect of this established provitamin A metabolism region across ancestries. Across both cohorts, although the lead variants differed, association signals colocalized to the same chromosome 16 region that spanning *PKD1L2* and the upstream regulatory neighborhood of *BCO1* (Figure 6).

As a *BCO1*-*PKD1L2* association was initially reported by others to be present among European genetic ancestry groups^42^, we evaluated the top two SNPs (rs4148211 located in *BCO1* and rs7194871 located in *CETP*) for association with β-carotene concentrations in all four ancestry groups. A nominally significant association was observed for rs4148211 in the Asian group (P=0.012, n=51), while no significant associations were found for rs7194871 (p>0.5).

### Rare, predicted-damaging variants are observed in individuals with extreme carotenoid concentrations

Rare, predicted-damaging variants in coding regions of genes can have large effects on physiologic traits^43^. We, therefore, looked for rare, putatively protein-damaging variants (**Supplementary Methods**) among individuals with extreme values (>2SD or <2SD) for either plasma carotenoids across the Primary Study Cohort (**Supplementary Table S8**).

In the Intervention Cohort, we identified an individual carrying a rare, predicteddamaging, missense variant in the *BCO1* gene (rs142824860, p.Glu14Gly; gnomAD MAF = 9.7 × 10^−5^; CADD score 25.0) (**Supplementary Methods**). This individual also had the lowest plasma β-carotene concentrations in the cohort (**Supplementary Table S9**). Among extreme plasma carotenoid phenotypes, we also observed rare or ancestry-enriched missense variants in lipid-related genes, including *CETP* (rs2303790, p.Asp459Gly; gnomAD MAF = 0.002), enriched among individuals of East Asian ancestry, and *APOA1* (rs756535387; p.Arg201Ser; CADD score 24.0), enriched among participants with markedly elevated plasma β-cryptoxanthin (**Supplementary Table S9**). These observations support a mechanistic intersection between carotenoid handling and lipoprotein biology and motivate follow-up in larger sequencing studies. These outlier observations were visualized by plotting plasma β-cryptoxanthin concentration against skin carotenoid status and highlighting prioritized rare-variant carriers (Figure 7).

## DISCUSSION

In this multi-ancestry nutrigenetics study, we used plasma carotenoid concentrations as integrated phenotypes of dietary phytochemical metabolism to understand how genetic ancestry, genetic variation, and genotype-by-diet interactions contribute to carotenoid status. Three findings define the study. First, SNP-based heritability was species-specific and host ancestry-dependent, with β-cryptoxanthin showing the highest estimate among measured carotenoids. Second, GWAS identified β-cryptoxanthin-associated loci at 3q13 and 19p13, with cross-cohort support for the 3q13 locus. Third, in a randomized dietary intervention, 3q13 variants modified β-cryptoxanthin response to carotenoid supplementation. Together, these findings support β-cryptoxanthin as a genetically responsive carotenoid phenotype and demonstrate the value of combining multi-ancestry genetics with controlled dietary intervention to study nutrient metabolism. This finding highlights the value of combining multi-ancestry genetics with controlled dietary intervention to study metabolic heterogeneity and differential dietary responses across individuals^1–4^.

The heritability of plasma carotenoid concentrations varied substantially by carotenoid species and across ancestry clusters, suggesting a more nuanced interpretation of plasma carotenoid concentrations may be warranted at the individual level. Total carotenoids showed low SNP-based heritability, consistent with the fact that the total measurement aggregates multiple carotenoid species and physiological contributors of carotenoid metabolism, such as intestinal absorption, lipoprotein transport, and enzymatic conversion, differ in their sensitivity to genetic variation^7,11,24,44^. In contrast, the magnitude of β-cryptoxanthin heritability is the highest among measured species, suggesting that this carotenoid is sensitive to genetically mediated differences in absorption, transport, and conversion. These findings extend prior evidence that circulating carotenoid concentrations show ancestry-dependent differences in relative heritability, including elevated heritability estimates in AMR-clustering participants across multiple carotenoid species, consistent with prior evidence for high heritability of plasma carotenoid in Mexican American populations^45^. Together, these results suggest that both genetic architecture and the balance of genetic versus environmental variance can differ across populations, reinforcing the need to study nutrient phenotypes in diverse cohorts^5,6,14,15^.

Consistent with β-cryptoxanthin’s elevated heritability, we identified association signals at 3q13 and 19p13 for β-cryptoxanthin that were supported across cohorts, together explaining a substantial fraction of phenotypic variance in the discovery cohort. While β-cryptoxanthin has been comparatively under-studied genetically, it is a bioavailable provitamin A carotenoid with^7,46^. The 19p13 signal overlaps regulatory candidates including *MED16/R3HDM4*, and the lead variant is an eQTL for *MED16* in multiple tissues, supporting a hypothesis of transcriptional regulation relevant to lipid- and retinoid-linked pathways^47–50^. MED16 encodes a subunit if the Mediator complex, which coordinates RNA polymerase II transcription and participates in nuclear receptor dependent transcriptional regulation^51^.

The randomized Intervention Cohort provides an important extension beyond cross-sectional association. Observational plasma concentration associations cannot distinguish genetic effects on habitual dietary exposure from genetic effects on carotenoid absorption, transport, conversion, or accumulation. By testing β-cryptoxanthin response under randomized supplementation and adjusting for baseline concentrations, we directly evaluated genotype-dependent response under defined dietary exposure, indicating a genotype-dependent component of carotenoid accumulation under controlled intake. Although the genotype-by-dose signals require confirmation in larger trials, this finding moves beyond static association toward actionable gene–diet biology, implying that β-cryptoxanthin levels are highly dependent upon an individual’s genetically-mediated metabolism which differs by their level of dietary intake, such that the effect of a certain dietary intake level of β-cryptoxanthin would depend on the individual’s underlying genotype; such observations are essential in the movement toward precision nutrition.

Targeted sequencing complemented array-based discovery by improving coverage at established carotenoid metabolism regions that are often incompletely captured by fixed-content arrays in genetically diverse populations^23,40^. At the *PKD1L2–BCO1* locus, sequencing-supported associations with β-carotene are consistent with prior work implicating this region in provitamin A metabolism and carotenoid status and highlight the challenges of pinpointing causal variants across ancestries with differing linkage disequilibrium patterns^12,13,41^. Finally, outlier-based analyses identified rare predicted damaging variants in carotenoid- and lipid-related genes (including *BCO1*, *CETP*, and *APOA1*), consistent with evidence that rare coding variation can exert large effects on metabolic traits and further supporting a mechanistic intersection between carotenoid handling and lipid transport^43^.

These findings should be interpreted in the context of several study limitations. First, the sample size is modest for GWAS, ancestry-stratified heritability and genotype-by-dose interaction analyses, however the small sample size is offset by strengths that are uncommon in larger population GWAS, including HPLC-based carotenoid phenotyping, repeated dietary measurement, longitudinal randomized supplementation, multi-ancestry cohort recruitment, replication in a non-overlapping intervention subset, and targeted sequencing of carotenoid metabolism genes. Nonetheless, we had limited power to detect smaller-effect loci, subgroup heritability estimates had large standard errors, and the variance explained by lead loci may also be inflated, at least in the discovery cohort. Second, although carotenoid exposure was highly controlled during the randomized intervention, dietary intake outside the intervention is still subjected to measurement error; interaction results are thus reported at nominal thresholds. Third, array genotyping and imputation in diverse cohorts can miss rare or ancestry-specific variation in our highly diverse cohort, even with high depth targeted sequencing at selected candidate genes. Lastly, further external replication is constrained by the limited availability of larger cohorts with harmonized plasma carotenoid profiling including multi-species, multi-ancestral cohort, detailed dietary measurements and intervention-based phenotyping. Larger multi-ancestry studies integrating deeper sequencing, local ancestry-aware methods and controlled dietary exposure will be important for fine-mapping these loci and and testing the mechanisms underlying genotype–diet interaction in circulating carotenoids^14,15,52^.

In summary, this study identifies species-specific and ancestry-dependent genetic contributions to plasma carotenoid phenotypes and provides evidence that common genetic variation can modify β-cryptoxanthin response to controlled carotenoid intake. By integrating genome-wide discovery, randomized dietary intervention phenotyping, and target sequencing, we demonstrate a tractable framework for moving gene-nutrient studies from static biomarker association toward experimentally anchored models of diet-responsive nutrient metabolism. These findings suggest that genetic variation may contribute to both population-level differences and individual outliers in plasma carotenoid concentrations, particularly for carotenoid species under stronger genetic control. More broadly, they indicate that plasma carotenoid concentrations should not be interpreted solely as biomarkers of intake, but as integrated phenotypes shaped by diet, ancestry, and genetically mediated metabolism. Although these results are not yet sufficient to guide genotype-based dietary recommendations in clinic practice, they provide a critical framework for future precision nutrition studies that integrate multi-ancestry genetics with controlled dietary intervention.

## Figures and Tables

**Figure 1.**
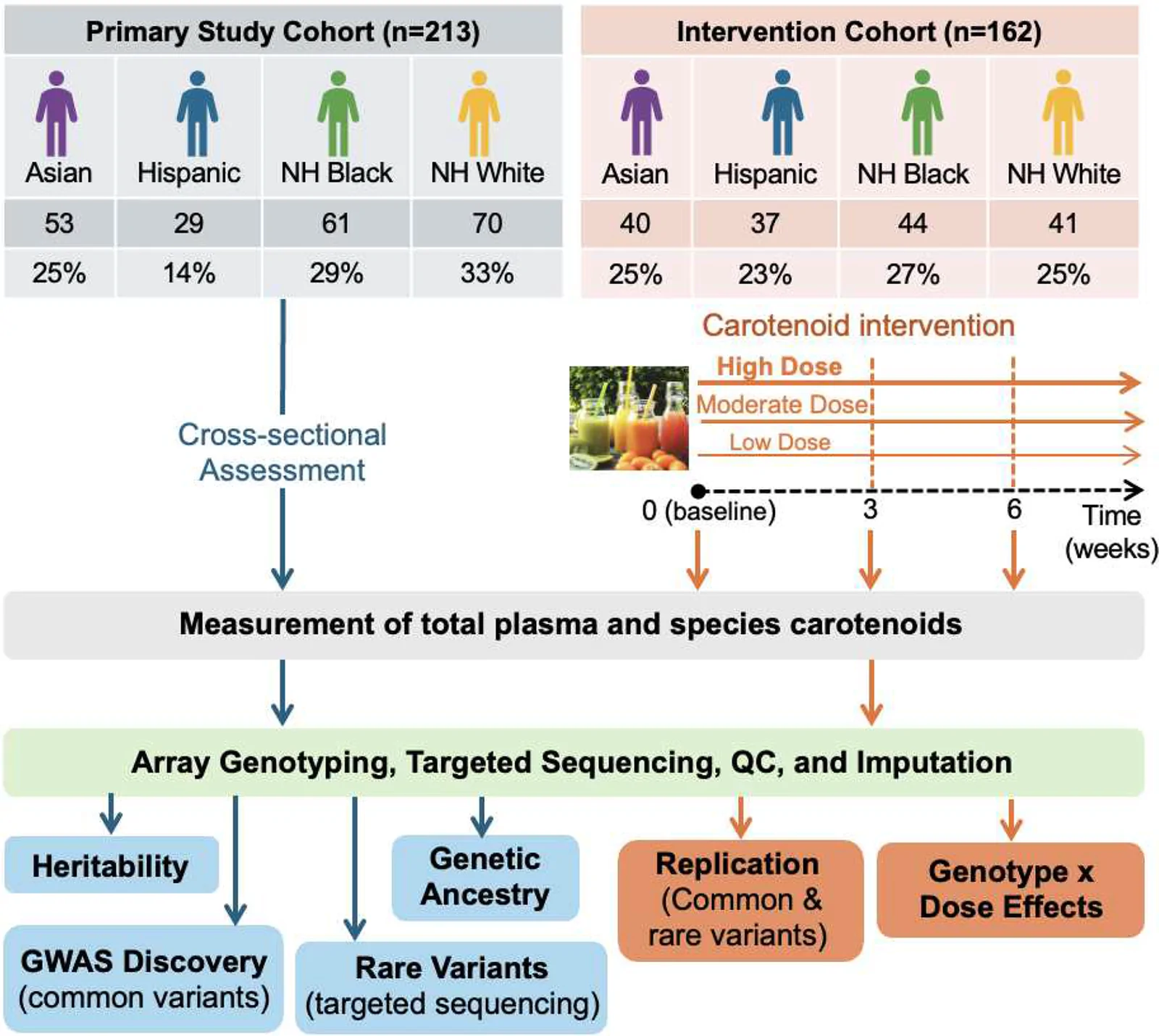
Overview of study design and analysis workflow. The study includes participants from two cohorts: a **Primary Study Cohort** (n=213) used for discovery analyses and an **Intervention Cohort** (n=162) used for replication and genotype-by dose analyses. Both comprised of multiple racial/ethnic groups (Asian, Hispanic, non-Hispanic Black, and non-Hispanic White), with sample sizes and proportions detailed in the top panel. The Intervention Cohort was randomized to receive a daily carotenoid-rich juice providing 0 mg/day (Control), 4 mg/day (moderate), or 8 mg/day (high) total carotenoids, with plasma carotenoids measured at baseline and at week 3 and week 6. Data were collected from previous studies^16,17^. Array genotyping data were used to infer genetic ancestry, estimate heritability, and perform GWAS to identify common variants, and test genotype-by-dose effects in the intervention cohort. Targeted sequencing was used to improve coverage of candidate carotenoid metabolism genes and to evaluate rare variants among individuals with extreme carotenoid phenotypes.

**Figure 2.**
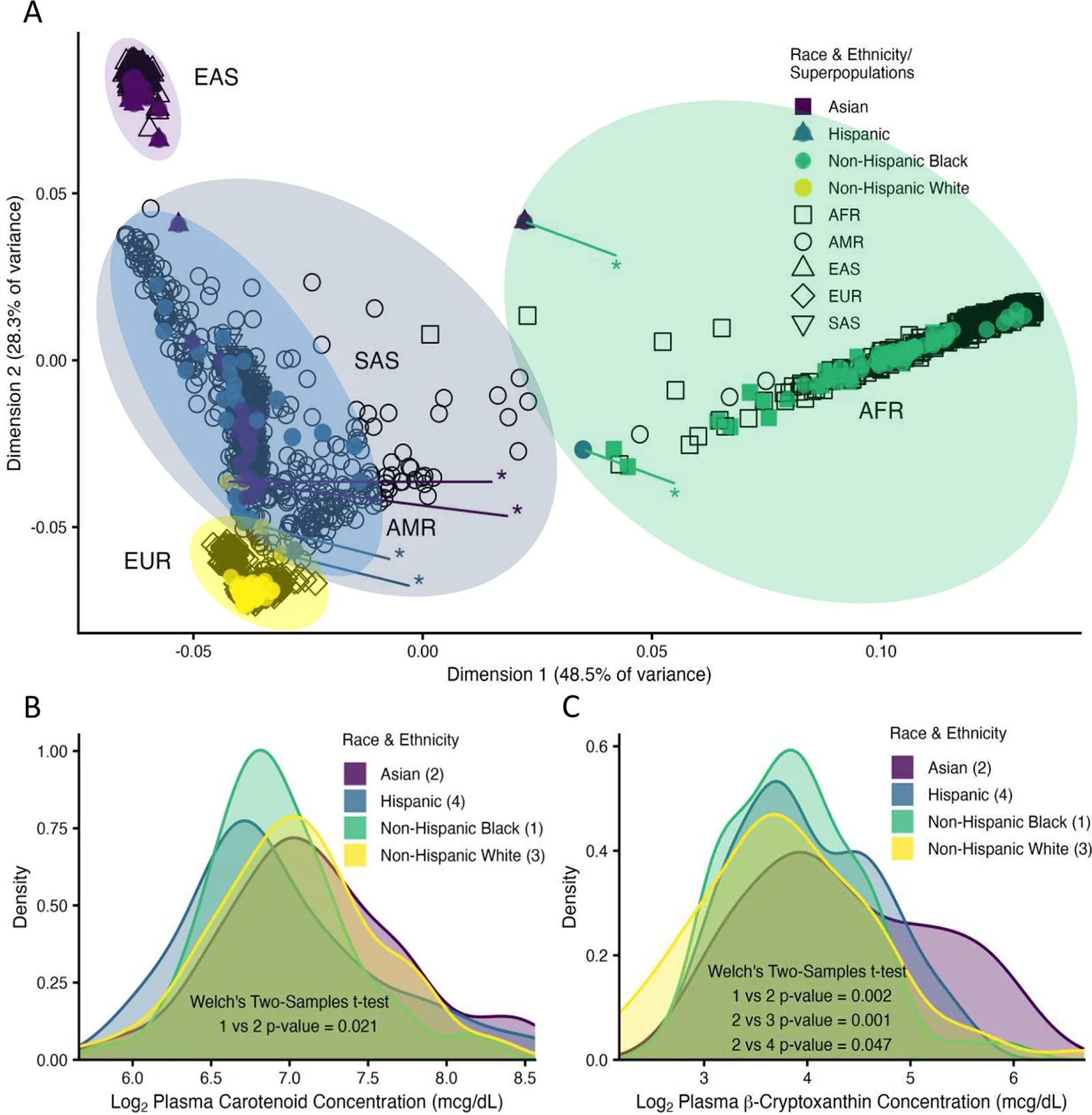
Self-reported race/ethnicity, genetically inferred ancestry, and plasma carotenoid concentrations in the Primary Study Cohort. Self-reported race/ethnicity groups include Asian (n=53), Hispanic (n=29), Non-Hispanic Black (n=61), and Non-Hispanic White (n=70). **2A**, Multidimensional Scaling (MDS) of cohort genotypes alongside 1000 Genomes Project reference superpopulations: AFR, African; AMR, Admixed American; EAS, East Asian; EUR, European; SAS, South Asian. Asterisks indicate individuals whose self-reported racial/ethnic group did not align with the majority genetic ancestry cluster for that group. **2B**, total plasma carotenoid concentration. **2C**, plasma β-cryptoxanthin concentrations. Concentrations are shown as log_2_-transformed mcg/dL. Group differences in panels B and C were assessed using unadjusted Welch’s two-sample t-test and presented descriptively.

**Figure 3.**
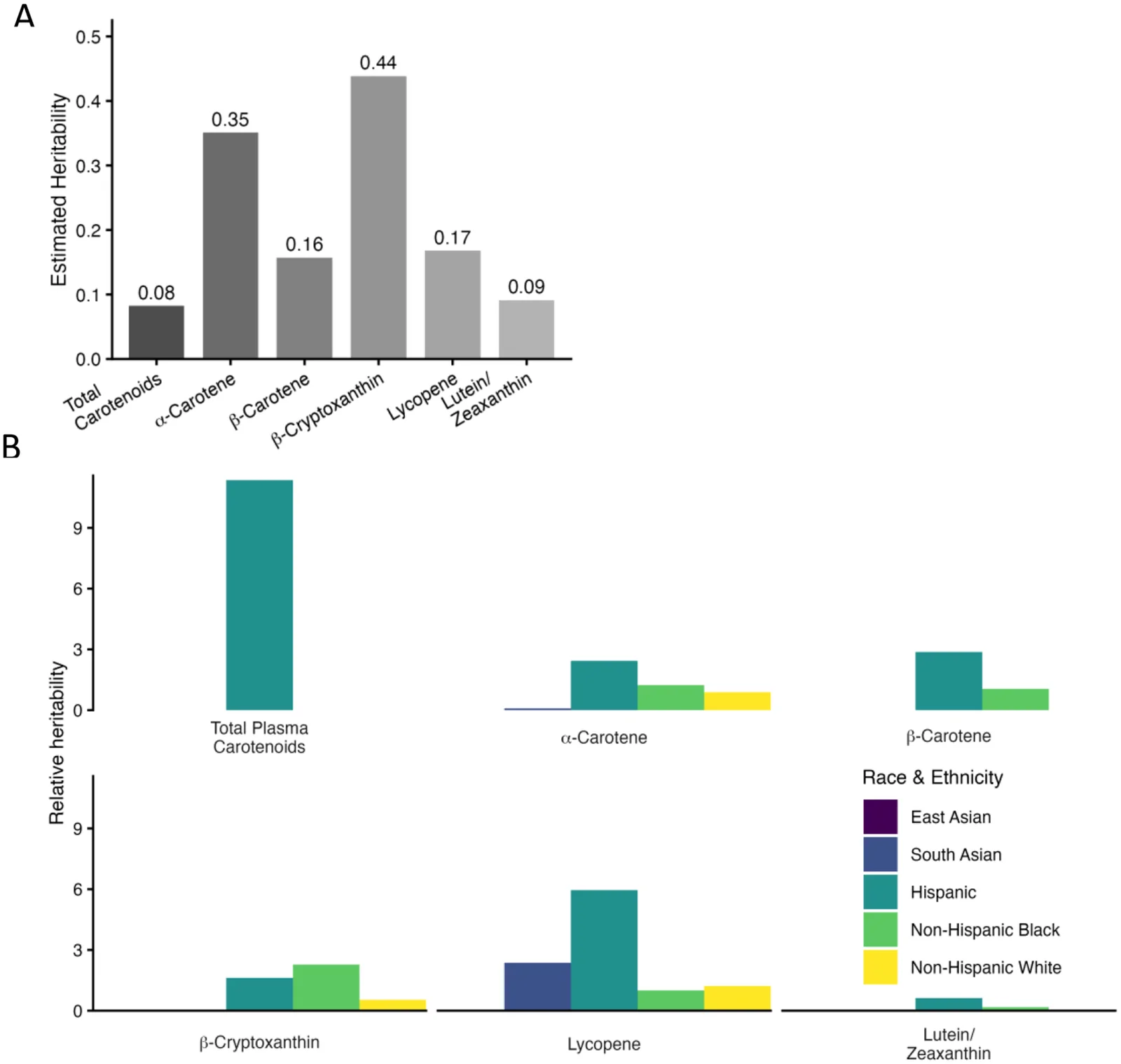
SNP-based heritability of plasma carotenoids. **3A**, SNP-based heritability estimates for total and species-specific plasma carotenoid concentrations across the entire Primary Study Cohort. **3B**, relative heritability estimation by genetically inferred ancestry groups. Genetic ancestry is defined based on multidimensional scaling (MDS) analysis. Relative heritability is calculated as the heritability estimate in each subgroup divided by the heritability estimate for the entire Primary Study Cohort for each carotenoid species.

**Figure 4.**
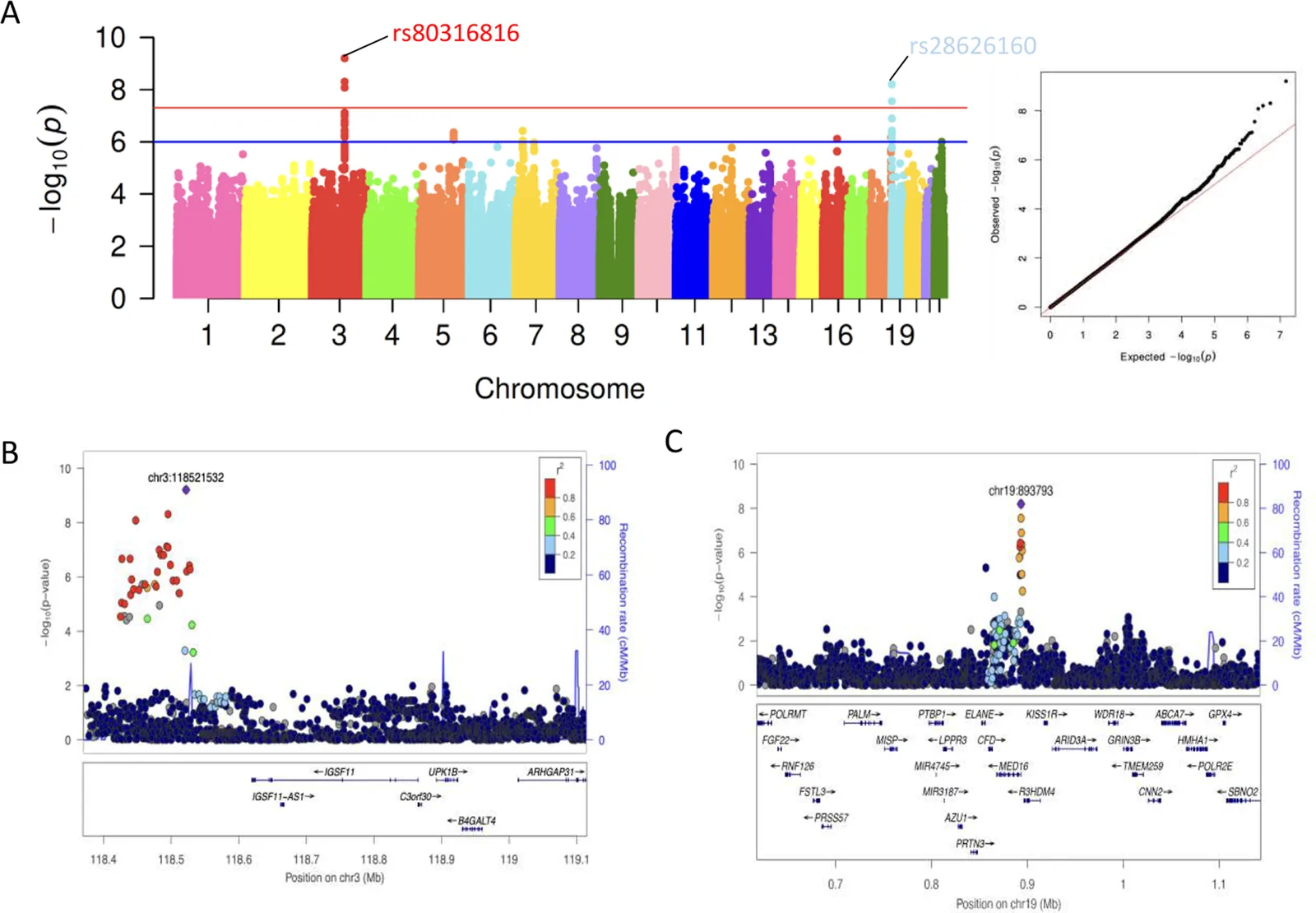
Genome-wide association identifies β-cryptoxanthin associated loci at 3q13 and 19p13. **A**. Manhattan plot of genome-wide association results for log_2_-transformed plasma β-cryptoxanthin concentration in the Primary Study Cohort using GEMMA linear mixed model. The red horizontal line indicates genome-wide significance (P < 5×10^−8^) and the blue line indicates the suggestive threshold (P < 1×10^−6^). The inset shows the corresponding quantile–quantile (QQ) plot. **B**. regional association plot for the 3q13 locus near *IGSF11*, highlighting the lead variants rs80316816 as the led variant. **C**. regional association plot for the 19p13 locus near *R3HDM4/MED16*, highlighting the lead variant rs28468554 as lead variant. In regional plots, the purple diamond denotes the lead SNP, point colors indicate linkage disequilibrium (r^2^) with the lead SNP, and the blue curve indicates local recombination rate. Genome coordinates are shown on the GRCh37/hg19 build.

**Figure 5.**
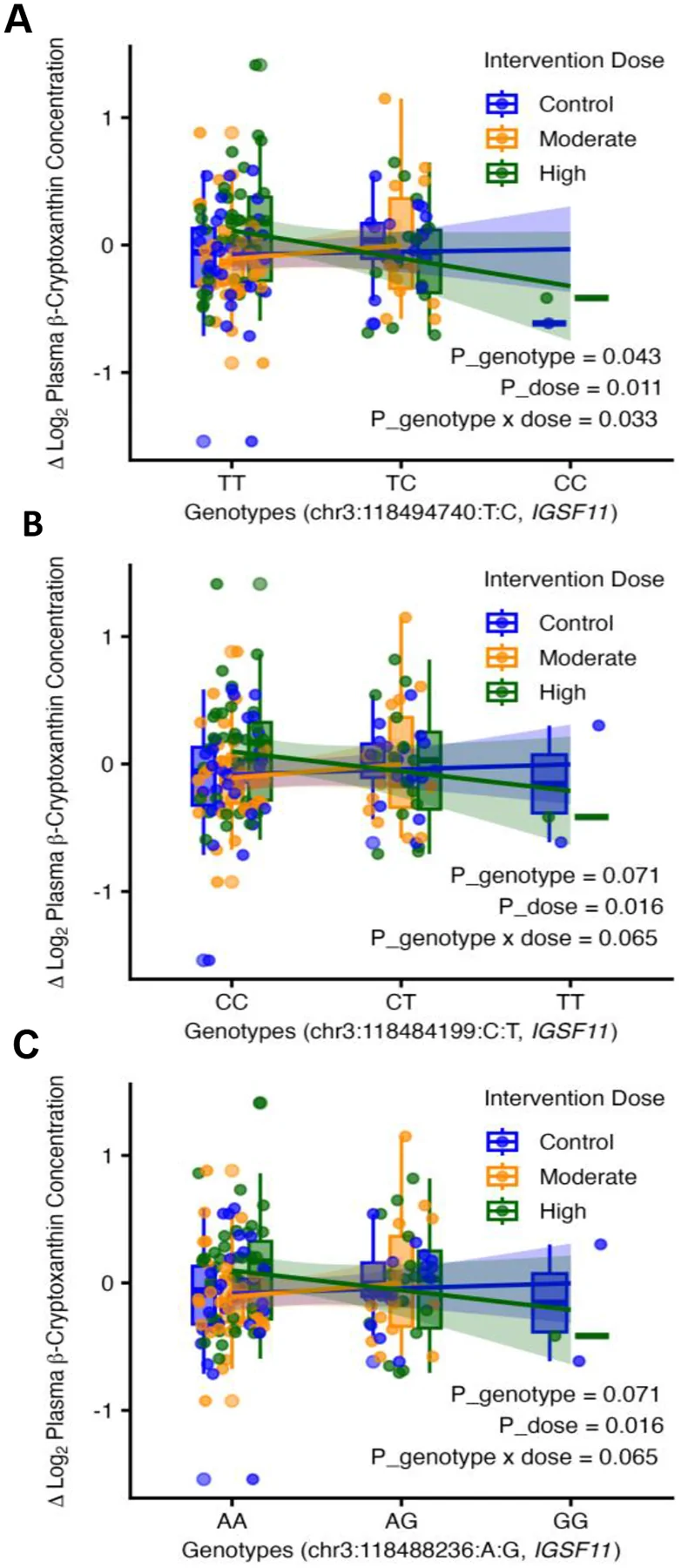
Genotype-by-dose interaction for plasma β-cryptoxanthin response across selected 3q13 loci in a 6-week randomized dietary carotenoid dose–response trial. Change in plasma β-cryptoxanthin concentration from Week 0 to Week 6 (Δlog_2_-transformed) is shown by genotype (x-axis; additive genotype categories) and randomized dose group (color). Dose groups were Control (0 mg total carotenoids/day), Moderate (4 mg/day) and High (8 mg/day). Panels correspond to tested loci at 3q13 near *IGSF11*: **A**. chr3:118494740 T>C; **B**. chr3:118484199 C>T; **C**. chr3:118488236 A>G. Boxplots summarize the distribution within each genotype–dose stratum (center line, median; box, interquartile range (IQR); whiskers, 1.5 × IQR). Lines represent fitted trends within each dose group to illustrate genotype-dependent differences in response to supplementation. P values correspond to the main effects of genotype (P_genotype), dose (P_dose), and the genotype × dose interaction (P_genotype×dose) from the interaction model described in Methods. Loci were prioritized for visualization when P_genotype×dose < 0.1, reflecting the exploratory nature of interaction testing in this cohort.

**Figure 6.**
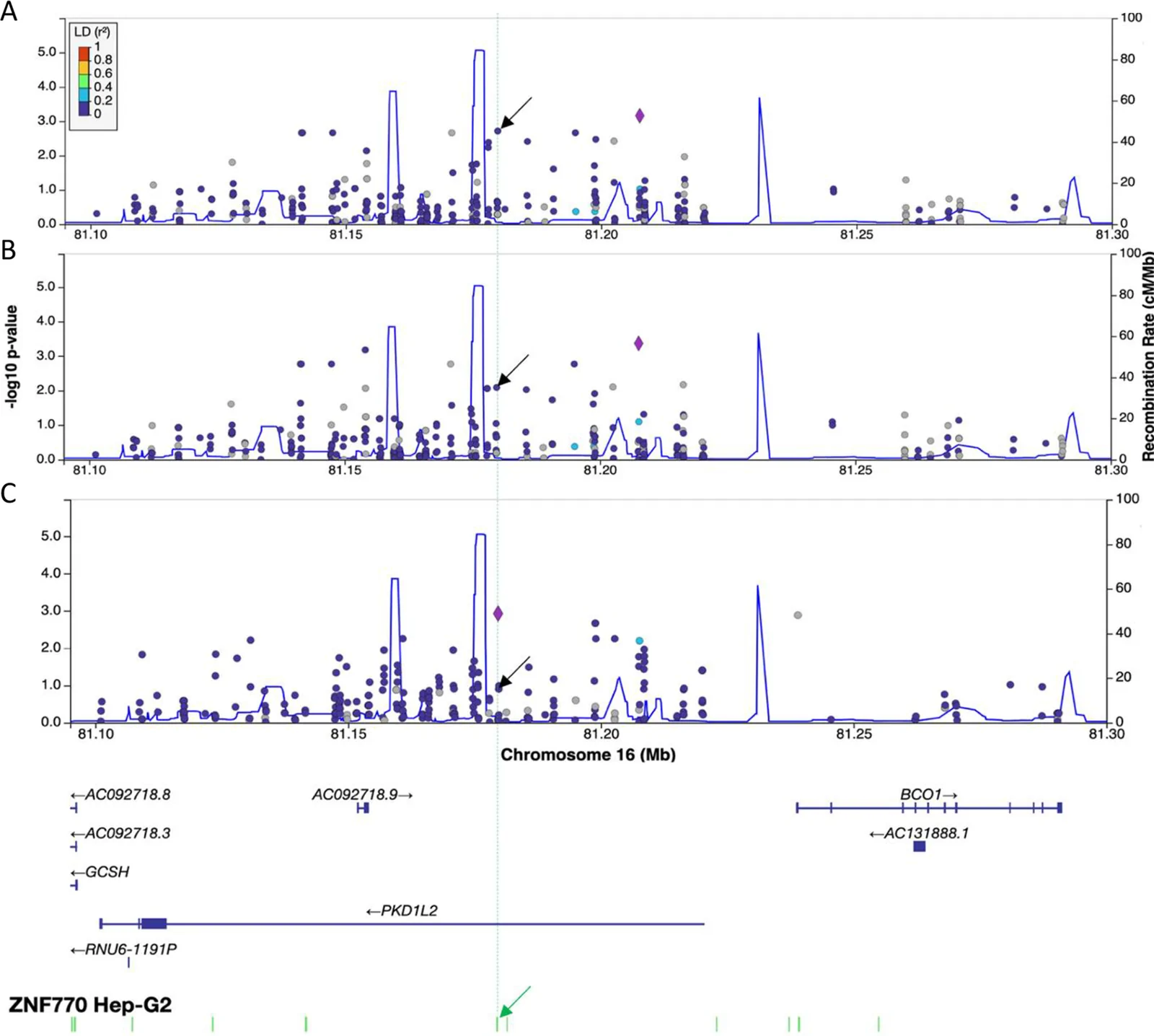
Targeted sequencing association with the *PKD1L2-BCO1 locus* for β-carotene. Regional association plots show targeted sequencing variants at the *PKD1L2-BCO1* locus associated with plasma β-carotene concentration. The y-axis displays the −log_10_P, and the x-axis displays genomic coordinates on GRCh38. **A**. Primary Study Cohort association analysis adjusted for standard covariates, with rs79139155 as the lead variant. **B**. sensitive analysis replacing MDS dimensions, also highlighting rs79139155. **C**. Intervention cohort replication analysis, highlighting rs4889261. Top association signals are represented by a diamond shape. Point colors indicate LD (r^2^) with the lead variant, and the dotted vertical line marks the overlap of rs4889261 (black arrows) with ZNF770 ChIP-Seq regulatory sites in Hep-G2 cells (Green arrow).

**Figure 7.**
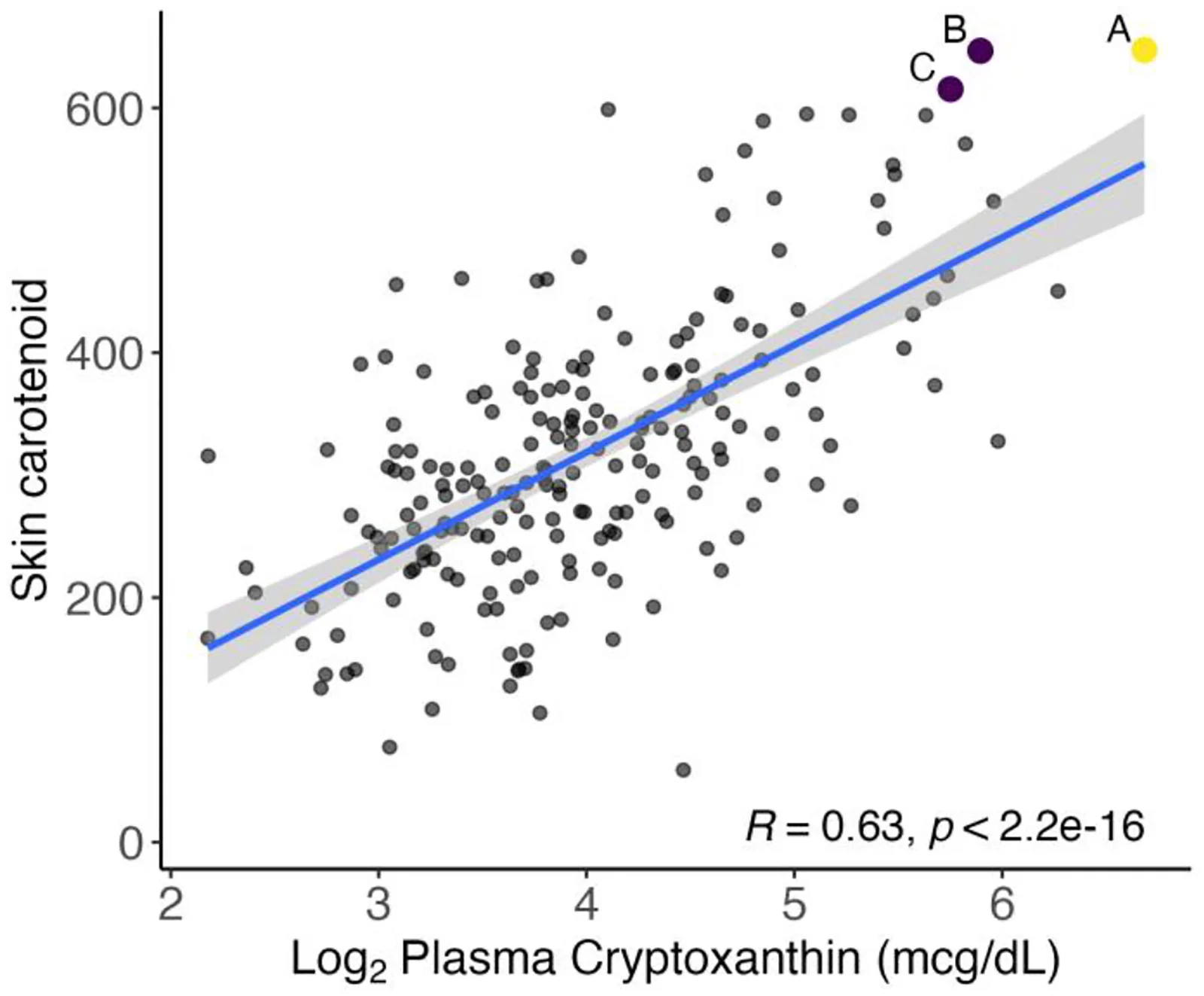
Rare predicted-damaging variants among individuals with extreme plasma carotenoid phenotypes. Plasma β-cryptoxanthin concentration is plotted against skin carotenoid score. Individuals carrying prioritized rare variants are highlighted and labeled as Supplementary Table S9. Colors indicated race-ethnicity of the individuals: yellow, Non-Hispanic White; dark purple, Asian. The blue line and shaded band represent the fitted linear regression and 95% confidence interval.

**Table 1.** Lead SNPs associations for plasma carotenoid phenotypes in discovery GWAS and cross-cohort meta-analysis. SNPs are reported as chr:pos:ref:alt. AF, minor allele frequency in the discovery cohort. Beta, per allele effect size from GEMMA linear mixed model (log2 concentration units); P, p-value from the Primary Study Cohort GWAS; P_meta, p-value from cross-cohort meta-analysis (METAL). SNPs in bold were directly genotyped on the Infinium^™^ H3Africa Consortium Array v2; the remaining SNPs were imputed.

Phenotype	Nearest Gene	SNPs (chr:pos:ref:alt)	rsID	AF	Beta	P	P_meta
**α-Carotene**	***RAPGEF1***	**9:134458148:G:A**	**rs3765544**	**0.054**	**0.75**	**8.86E-10**	**1.74E-09**
**β-Carotene**	***RAPGEF1***	**9:134458148:G:A**	**rs3765544**	**0.054**	**0.79**	**6.43E-08**	**1.01E-07**
β-Cryptoxanthin	*igsf11*	3:118446886:T:C	rs76087842	0.08	0.72	8.32E-09	1.32E-06
**β-Cryptoxanthin**	***IGSF11***	**3:118492749:G:A**	**rs798600**	**0.129**	**0.55**	**7.63E-08**	**3.52E-06**
β-Cryptoxanthin	*IGSF11*	3:118494728:C:G	rs76613159	0.078	0.71	4.95E-09	1.62E-08
β-Cryptoxanthin	*IGSF11*	3:118494740:T:C	rs1088589	0.137	0.53	8.21E-08	1.08E-04
β-Cryptoxanthin	*IGSF11*	3:118521532:T:C	rs80316816	0.08	0.74	6.25E-10	1.34E-06
β-Cryptoxanthin	*R3HDM4*	19:893793:C:A	rs28468554	0.359	−0.46	6.26E-09	1.76E-06
**β-Cryptoxanthin**	***R3HDM4***	**19:893910:A:G**	**rs28626160**	**0.495**	**−0.37**	**2.77E-08**	**1.96E-05**

## Data Availability

H3Africa Array - https://h3africa.org/index.php/2019/12/12/h3africa-chip-faq/ H3Africa Array Data Sheet - https://www.illumina.com/downloads/infinium-h3africa-consortium-array-data-sheet-370-2020-001.html 1000 Genomes Project Resource - https://www.internationalgenome.org/ Individual-level genotype, dietary, and plasma carotenoid data are subject to participant consent and institutional data-use restrictions. De-identified summary statistics, source data underlying the main figures, and targeted sequencing variant-level association results will be available from the corresponding author upon reasonable request. Custom scripts used for genotype processing, association analyses, heritability estimation, genotype-by-dose interaction testing, and figure generation are available from the corresponding author for peer review. No new software package was developed for this study.
